# Negative life events, sleep quality and depression in university students

**DOI:** 10.1038/s41598-025-08635-6

**Published:** 2025-07-01

**Authors:** Zhe Zhang, Zimo Cai, Qian Meng

**Affiliations:** College of Education, Bohai University, 19 Keji Road, Jinzhou, 121013 China

**Keywords:** University students, Negative life events, Depression, Sleep quality, Psychology, Health care

## Abstract

The rate of depression among university students is increasing. University students experiencing negative life events are at risk of developing depression, which could further decrease their academic engagement. In addition, negative life events are often accompanied by sleep problems. This study used a cross-sectional design to explore the complex relationships among negative life events, sleep quality, and depression in university students, with a particular focus on whether sleep quality serves as a mediating factor in the association between negative life events and depression. Three self-report scales were completed by 828 participants recruited from three universities. The results revealed that negative life events and sleep quality had a significant effect on depression separately and that sleep quality played a mediating role in the relationship between negative life events and depression in university students. Our study contributes to a deeper understanding of the relationships among negative life events, sleep quality, and depression and has important implications for the prevention and intervention of depressive symptoms in university students. By acknowledging the role of negative life events, educators and counselors can be more proactive in identifying students who may be struggling and providing them with the necessary support. By understanding the mediating role of sleep quality, university students can more effectively recognize and manage their mental health needs and cultivate resilience strategies to handle stressors to reduce their risk of developing depressive symptoms.

## Introduction

Depressive disorder (also known as depression) is a common mental disorder. According to the World Health Organization, depression involves a depressed mood (feeling sad, irritable, or empty) or a loss of pleasure or interest in activities for long periods of time^1^. A person experiencing depression typically exhibits several of the following symptoms: feelings of sadness, hopelessness, or pessimism; decreased self-esteem and increased self-criticism; a diminished or lack of ability to take pleasure in everyday activities; reduced energy and vitality; appetite loss; and disrupted sleep or insomnia^2^. Depression can affect anyone regardless of age, gender, race or ethnicity, income, culture, or education level^3^, including university students. University students, who are in a transitional stage of life, are more prone to mental health issues such as depression and anxiety^4^. The rate of depression among university students is increasing. The 2021 Winter/Spring Data Report from the annual Healthy Minds Study, which was based on an online survey of 96,000 students across 133 campuses in the U.S. during the 2021–22 academic year, revealed that 44% of the students reported experiencing symptoms of depression, 37% reported having anxiety disorders, and 15% reported having seriously considered suicide in the past year; these rates were the highest recorded in the history of the 15-year survey^5^. Negative life events and adverse environmental factors, including factors such as poor academic performance, interpersonal problems and concerns about future employment, have been shown to predict increases in depressive symptoms^6^ and the onset of major depression in university students^7^. University students experiencing negative life events are at risk of developing depression, which could further decrease their academic engagement^8^. Moreover, previous studies have suggested that there is a strong association between sleep quality and depression or depressive symptoms^9–12^.

However, negative life events are often accompanied by sleep problems^13^. Therefore, exploring the possible mediating role of sleep quality in the relationship between negative life events and depression in university students is highly important for a deeper understanding of the mechanism of depression and the development of effective intervention strategies. This study aimed to explore the effect of negative life events on depression among university students, with a focus on the mediating role of sleep quality. Our study adds to the body of literature concerning the link between negative life events and depression in university students and attempted to pinpoint the mediating role of sleep quality, which has been overlooked by other studies. This was a nonclinical study that specifically focused on subclinical levels of depression among university students. This study concentrated on the continuum of depressive symptoms rather than clinical diagnoses, enabling the exploration of how negative life events and sleep disturbances contribute to depressive symptoms in a nonclinical population, which has significant implications for early intervention and prevention strategies.

## Literature review

### Negative life events and mental health

On the basis of pioneering research by Brown & Harris^14,15^, it is now widely accepted that stressful life events play a critical role in the development of depressive symptoms and clinical depression. Furthermore, the strong correlation between depression and adverse life events among students worldwide is well documented^16^. Negative life events include events related to interpersonal relationships, academic stress, health problems, adjustment problems, and other stressors^17^. Negative life events are also major causes of recurrent depression and direct triggers of depression. A 9-year longitudinal study revealed that negative life events increased the risk of depressive symptoms in individuals^18^.

The university period is a crucial stage in life, during which college students face pressure from academics, interpersonal relationships, future development, and other aspects, which may have an impact on their mental health. A study of 3629 college students revealed that those who experienced more negative life events were more likely to have more severe depressive symptoms^8^. The aforementioned studies indicate that negative life events can trigger different severities of depressive symptoms among college students. If left untreated, their depressive symptoms may escalate, posing a risk of self-harm among the affected students^19^. The relationship between negative life events and depression can potentially decrease the academic engagement and academic performance of college students.

### Sleep quality and mental health

Sleep, a natural and recurrent state of the body used to rest and repair itself, has been widely studied for its profound effect on cognitive function^20^, emotional regulation^21^, and overall mental health^22^. Sleep quality and mental health go hand-in-hand, with many, if not all, mental health problems associated with sleep problems^23^. Insufficient sleep can impair an individual’s capacity to perform daily functions and may have significant ramifications for both physical and mental wellbeing. Sleep problems are particularly common in individuals with anxiety, depression, bipolar disorder, and attention deficit hyperactivity disorder (ADHD)^24^.

Good sleep is crucial for maintaining good health. Sleep quality can influence both the onset and trajectory of a variety of mental health difficulties^23^. Studies on neurobiology indicate that poor sleep can disrupt the balance of neurotransmitters such as serotonin, dopamine, and norepinephrine and lead to hyperactivity of the hypothalamic‒pituitary‒adrenal (HPA) axis, resulting in elevated cortisol levels^25–27^. These are the key factors in the development of depression. For example, people who experience insomnia face a tenfold increased risk of developing depression compared with those who do not experience insomnia. Poor sleep can trigger mania, psychosis or paranoia or worsen existing symptoms. A lack of good-quality sleep can hinder the proper regulation of emotions, ultimately increasing individuals’ susceptibility to depression in the coming months or even years^28^.

In line with the existing research findings, the following hypotheses were proposed in this study:

H1: Negative life events have a significant effect on depression among university students.

H2: Sleep quality significantly correlates with depression among university students.

H3: Sleep quality plays a mediating role in the relationship between negative life events and depression in university students.

## Method

### Sample

All undergraduate students were recruited from three universities. We adopted a combination of online and offline methods to distribute questionnaires, depending on the preferences and accessibility of our target respondents, to ensure the validity and reliability of our research results. We posted recruitment notices for participants on university bulletin boards. Individuals who were interested in participating in the study were asked to contact us to complete the questionnaire. In addition, we requested the assistance of teachers to distribute the questionnaires during scheduled classes or seminar sessions. All participants were recruited from full-time undergraduate students, excluding part-time and international students, as our study aims to specifically investigate the relationships between negative life events, sleep, and depression among undergraduate students within the Chinese context.

Among the 900 participants, 828 completed the questionnaires, accounting for 92% of the total sample. In terms of gender, 45.89% of the students were female. In terms of years of study, 33.09% of the participants were sophomores, 29.11% were juniors, 24.03% were freshmen, and 13.77% were seniors. With respect to programs, 38.54% of the students were in social humanity programs, followed by engineering (34.80%) and natural science (26.66%) programs, as demonstrated in (Table 1).

**Table 1:** Descriptive statistics for categorical data.

Variables	*n*	%
Gender		
Male	448	54.11
Female	380	45.89
Years of study		
Freshman	199	24.03
Sophomore	274	33.09
Junior	241	29.11
Senior	114	13.77
Program		
Social humanity	315	38.05
Natural Science	274	33.09
Engineering	239	28.86

### Procedure

The study was conducted from March 20, 2024, to June 20, 2024. Before the participants completed the questionnaire, we provided them with a detailed explanation of the research purpose and confidentiality principles, and we ensured that they understood and agreed to participate voluntarily. Informed consent was obtained from all participants, ensuring their complete understanding of the purpose of the study and the confidentiality of their responses^29^. This research was approved by the Ethics Committee of Bohai University. The study adhered to the Declaration of Helsinki and subsequent amendments, and the ethical principles governing research involving human subjects were strictly followed.

### Measures

The participants were asked to complete three scales: the Negative Life Events scale (NLEs) designed by Liu^30^, the Pittsburgh Sleep Quality Index (PSQI) developed by Buysse^31^ and the Center for Epidemiologic Studies Depression Scale designed by Radloff (CES-D)^32^.

#### Negative life events scale (NLEs)

Xin^33^ updated and improved the Negative Life Events scale designed by Liu, making it more in line with the current living conditions of teenagers. The NLEs consists of 26 items with five factors: interpersonal stress (four items, e.g., conflict with classmates or friends), academic stress (four items, e.g., heavy load of study), punishment (seven items, e.g., being criticized or punished), loss (six items, e.g., conflict with the family), health and adaptation problems (five items, e.g., I don’t like school). The NLEs adopts a 6-point scoring method, where a score of 0 points represents no occurrence of the event, a score of 1 point represents no impact and a score of 5 points represents a severe impact. The NLEs items are scored on a scale ranging from 0 to 5 points. The higher the score is, the greater the impact of negative life events on university students. The Cronbach’s alpha coefficient of the NLEs was 0.936, and those of the interpersonal stress, academic pressure, punishment, loss, and health and adaptation problems subscales were 0.848, 0.840, 0.856, 0.886 and 0.916, respectively. The fit indices (χ2/df = 2.746, NFI = 0.936, GFI = 0.931, CFI = 0.958, and RMSEA = 0.046) indicate that the questionnaire has adequate reliability and validity. The composite reliability (CR) values for interpersonal stress, academic stress, punishment, loss, health and adaptation problems were 0.851, 0.842, 0.859, 0.887 and 0.916, respectively, and the average variance extracted (AVE) values for interpersonal stress, academic stress, punishment, loss, health and adaptation problems were 0.591, 0.572, 0.552, 0.567 and 0.610, respectively.

#### Pittsburgh sleep quality index (PSQI)

The Pittsburgh sleep quality index (PSQI), a short self-report questionnaire developed by Buysse, is the most widely used measure of sleep quality. The Chinese version of the PSQI was developed by Liu^34^ and consists of 24 questions that assess seven dimensions, scored on a scale ranging from 0 (best) to 3 (worst). These dimensions can be broadly grouped into sleep efficiency factors (sleep quality, sleep latency, sleep duration, and habitual sleep efficiency) and sleep disturbance factors (sleep disturbances, use of sleep medications, and daytime dysfunction). The total PSQI score ranges from 0 to 21 points, with scores of 0–4 points indicating good sleep and 7–21 points indicating bad sleep. The higher the PSQI score is, the worse an individual’s sleep quality. The Cronbach’s alpha coefficient of the PSQI was 0.90, and those of the sleep efficiency and sleep disturbance subscales were 0.883 and 0.909, respectively. The fit indices (χ2/df = 2.980, NFI = 0.990, GFI = 0.987, CFI = 0.993, and RMSEA = 0.049) indicate that the questionnaire has adequate reliability and validity. The composite reliability (CR) values for sleep efficiency and sleep disturbance were 0.886 and 0.909, respectively, and the average variance extracted (AVE) values for sleep efficiency and sleep disturbance were 0.661 and 0.768, respectively.

#### Center for epidemiologic studies-depression scale (CES-D)

Xin and Shen revised the Chinese version of the CES-D developed by Radloff, which is a 20-item self-report questionnaire designed to detect depression in the general population that covers four symptom areas, including depressed affect, anhedonia, somatic activity and interpersonal challenges^32^. The CES-D is rated on a four-point Likert scale (from 0 (rarely or none of the time) to 3 (most or all of the time)). The CES-D items are scored on a scale ranging from 0 to 5 points. The total score ranges from 0 to 60 points. Higher scores indicate a greater frequency of depressive symptoms. The Cronbach’s alpha coefficient of the CES-D was 0.924, and the coefficients of the subscales of depressed affect, anhedonia, somatic activity and interpersonal challenges were 0.941, 0.832, 0.882 and 0.843, respectively. The fit indices (χ2/df = 4.891, NFI = 0.927, GFI = 0.911, CFI = 0.941, and RMSEA = 0.069) indicate that the questionnaire has adequate reliability and validity. The composite reliability (CR) values for the depressed affect, anhedonia, somatic activity and interpersonal challenges subscales were 0.942, 0.843, 0.885 and 0.843, respectively, and the average variance extracted (AVE) values for the depressed affect, anhedonia, somatic activity and interpersonal challenges subscales were 0.669, 0.558, 0.566 and 0.729, respectively.

### Data analysis

**Fig. 1:**
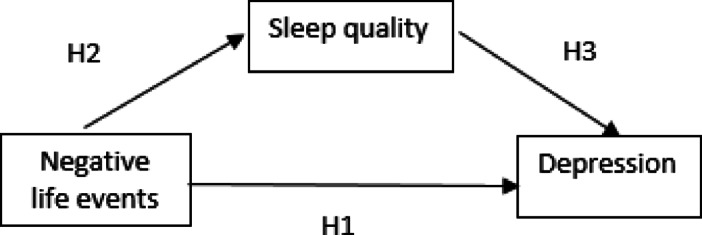
The estimated model.

The statistical correlations among negative life events, sleep quality, and depression among university students were tested through correlation analysis. We then utilized the Hayes PROCESS macro for SPSS, specifically Model 4, to further validate whether sleep quality functions as a mediator in the relationship between negative life events and depression among university students. The estimated model is demonstrated in (Fig. 1).The bootstrap method developed by Hayes^35^ is the most commonly used approach to evaluate mediation effects in the field of psychology as well as other fields. The significance of the direct path coefficients is tested using nonparametric confidence intervals obtained from 5,000 bootstrap resampling iterations. Mediation analysis serves as a potent tool for researchers aiming to unravel the complexities of relationships between variables, enabling them to comprehend the underlying mechanisms, pinpoint effective interventions, delve into intricate relationships, and enhance the reliability of study findings^36^.

## Results

### Correlation analyses

**Fig. 2:**
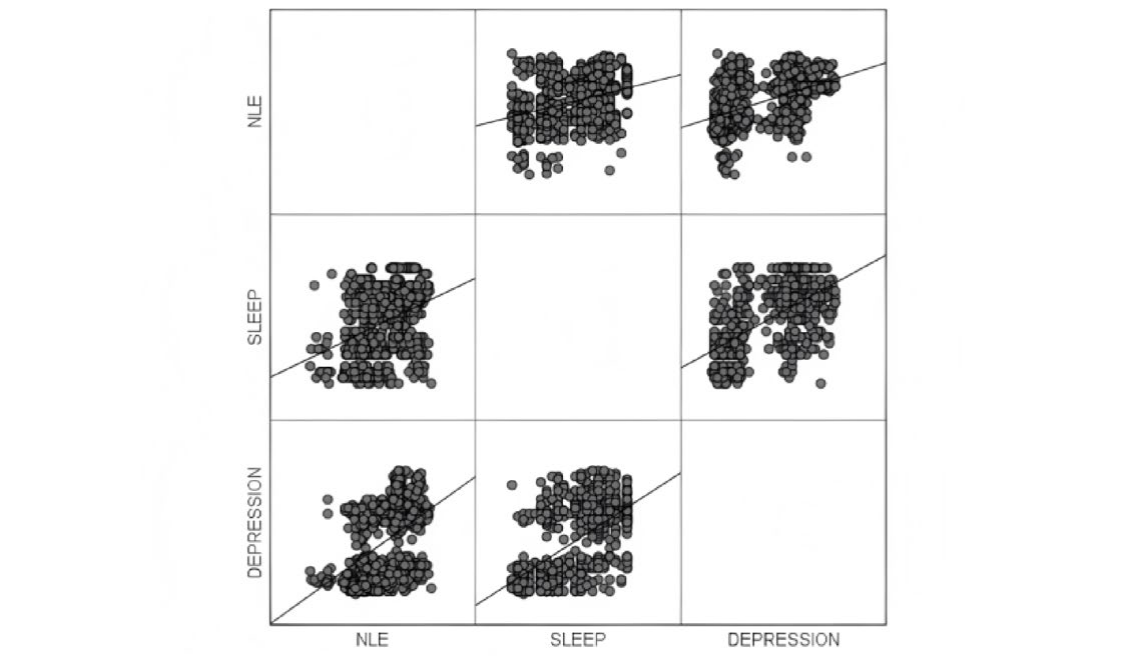
Scatter plots of negative life events, sleep quality, and depression.

The scatter plot visually demonstrated the correlations between negative life events, sleep quality, and depression (Fig. 2). Then correlation analysis was employed to further confirm the relationships between three variables. The means, standard deviations, and correlation matrices of negative life events, sleep quality, and depression are presented in (Table 2). The results from the correlation analysis indicate that there were significant correlations among negative life events, sleep quality, and depression. Negative life events were significantly correlated with depression (*r* = 0.477, *p* < 0.01), and sleep quality was significantly associated with depression (*r* = 0.597, *p* < 0.01). Moreover, there was a significant association between negative life events and sleep quality (*r* = 0.347, *p* < 0.01).

**Table 2:** Descriptive statistics and correlation analyses for the study variables.

	M	SD	1	2	3
Negative life events (1)	3.081	0.909	1		
Sleep quality (2)	11.531	5.855	0.347**	1	
Depression (3)	17.893	12.716	0.477**	0.597**	1

*p < 0.05 **p < 0.01.

### Regression analyses

As shown in Table 3, when controlling for gender, years of study and program, the regression analysis results revealed that negative life events could positively predict depression (β = 0.491, *p* < 0.01), which means that as the number of negative life events increases, the likelihood of depression also tends to increase. Moreover, sleep quality was significantly positively correlated with the level of depression, meaning that poor sleep may lead to or exacerbate depressive symptoms (β = 0.478, *p* < 0.01). Even when the mediating variable (sleep) was included in the regression model, the indirect influence of negative life events on depression remained notably significant (β = 0.305, *p* < 0.01).

**Table 3:** Results of regression analysis.

Variables	Dependent variable: Sleep quality		Dependent variable: depression			
	M1		M2		M3	
	Beta	t	Beta	t	Beta	t
Gender	0.072	2.196*	0.025	0.800	−0.011	−0.408
Years of study	−0.017	−0.509	0.017	0.546	0.025	0.952
Program	−0.007	−0.223	−0.002	−0.079	0.001	0.044
Negative life events	0.350	10.728***	0.478	15.572***	0.305	10.939***
Sleep quality					0.491	17.597***
R^2^	0.126		0.228		0.439	
∆R^2^	0.121		0.225		0.436	
F	29.576***		60.879***		128.895***	

*p < 0.05 **p < 0.01.

### Mediation analyses

To further explore the relationships among negative life events, sleep quality and depression, nonparametric bias-corrected bootstrapping was conducted to test the mediating effect of sleep quality on the relationship between negative life events and depression in university students. The results of the mediation analysis are presented in (Table 4). The total effect of negative life events on depression was 6.669, and the 95% confidence interval was [5.830, 7.509], indicating that the overall effect was valid. Regarding the direct effect, the 95% confidence interval (CI) of the direct effect of negative life events on depression was [3.529, 5.055], with a direct effect value of 4.292, constituting 64.36% of the total effect. Moreover, the nonparametric bootstrapping method confirmed the significance of the indirect effect of negative life events on depression mediated by sleep quality (95% bootstrap, CI = 1.883, 2.886). The value of the indirect effect of negative life events on depression through sleep quality was 2.377, accounting for 35.64% of the total effect.

**Table 4:** Results of the mediation analysis.

		Effect	SE	95% confidence Interval		Relative effect
				Lower	Upper	
Total effect	X→Y	6.669	0.428	5.830	7.509	–
Direct effect	X→Y	4.292	0.389	3.529	5.055	64.36%
Indirect effect	X→M→Y	2.377	0.254	1.883	2.886	35.64%

X: negative life events; Y: depression; M: sleep quality.

## Discussion

The results of the study confirm the association between negative life events and depression, supporting H1. The link between negative life events and depressive symptoms is well documented in the field of psychology^16^. Negative life events play a significant role in depression among university students. University students who experienced more negative life events were more likely to have elevated depressive symptoms^9^. When students face multiple stressors simultaneously, such as academic pressures, financial worries, and interpersonal conflicts, their stress levels can become overwhelming. Stressful life events are associated with an increased risk of mental disorders, a decreased quality of life and a decrease in academic success^19,37^. Additionally, negative life events can disrupt students’ sense of control and security^38^, contributing to feelings of despair and hopelessness, which are key features of depression^39^. Research suggests that negative life events can trigger changes in brain chemistry and neurobiology, leading to the development of depressive disorders^40^. Importantly, the impact of negative life events is often cumulative. Students who experience multiple negative events over time may be more likely to develop persistent depressive symptoms.

The results showed that sleep quality has a significant effect on depression among university students, confirming H2. Empirical research provides evidence of the correlation between sleep quality and depression among university students. Students with poorer sleep quality tend to have higher anxiety and depression scores^41^. Sleep is crucial for the restoration of brain function and memory consolidation^42^. Chronic sleep loss can lead to a decline in emotional regulation ability, making it more difficult for individuals to cope with negative emotions and pressures in life^25,27,28^. Furthermore, sleep deprivation may cause individuals to develop negative thinking^43^, which is the key component of depressive emotions^21,44^. Moreover, poor sleep can exacerbate the academic stress university students face and lead to poor academic performance, thereby increasing their risk of depression.

Correlation analysis shows a moderate link between negative life events, sleep, and depression. Previous research indicated that depression is a complex psychological phenomenon, influenced by a multitude of interrelated factors^10^. Psychological Factors (such as low self-esteem)^45^, physiological Factors (such as hormonal imbalances)^25^ and behavioral Factors( such as substance abuse)^46^. It is clear that we cannot rely solely on a simple linear correlation analysis to understand how negative life events, sleep, and depression are connected. However, our research findings nonetheless underscore the critical significance of negative life events and sleep in the context of depression.

The results of the mediation analysis revealed that sleep quality plays a mediating role in the relationship between negative life events and depression in university students, verifying H3. The study revealed that the life events of university students are significant predictors of their sleep quality and that the greater the number of negative life events experienced at school, especially those involving academic stress factors, the worse their sleep quality becomes^47^. The stress responses triggered by negative life events may disrupt the functioning of the autonomic nervous system, causing alterations in adrenocorticotropic hormone concentrations and cortisol levels, which may subsequently lead to abnormal sleep patterns^48^. Sleep deprivation impairs emotional regulation, increasing individuals’ susceptibility to anxiety, irritability, and other negative emotions, thereby increasing the risk of depression^22,23,49^. Therefore, we can conclude that sleep quality acts as a mediator of the relationship between negative life events and depression. Negative life events affect sleep quality, and a decrease in sleep quality further triggers or intensifies depressive symptoms.

### Implications

The findings of our study suggest that there are statistical relationships among negative life events, sleep quality, and depression among university students. Given these correlations, we can argue that negative life events and sleep quality are important factors to consider when studying depression among university students. By acknowledging the role of negative life events, educators and counselors can be more proactive in identifying students who may be struggling and providing them with the necessary support.

The results of the mediation test revealed that poor sleep quality can exacerbate the effect of negative life events on depression. These findings suggest that improving sleep hygiene and addressing sleep disturbances could be effective strategies for reducing depression among university students. These results suggest that improving sleep quality may help alleviate depressive symptoms caused by negative life events. For example, establishing good sleep habits, creating a comfortable sleep environment, and using other methods to enhance sleep quality may help individuals better cope with the psychological pressure caused by negative life events^29^.

By understanding the interplay among negative life events, sleep quality and depression, university students can more effectively recognize and manage their mental health needs and cultivate resilience strategies to handle stressors^50^. In universities and education, it is important to recognize the risks of negative life events and provide appropriate support to help students cope with negative life events and reduce their risk of developing depressive symptoms^8^.

## Conclusion

This study aimed to explore the complex relationships among negative life events, sleep quality, and depression in university students, with a particular focus on whether sleep quality serves as a mediating factor in the association between negative life events and depression in university students. Our study provides a comprehensive understanding of how sleep quality functions as a mediator in the relationship between negative life events and depression, which has been ignored by previous studies among university students. Our study underscores the importance of sleep in maintaining well-being and early intervention in reducing the risk of negative life events.

Although novel, our study is not without limitations. First, our study used a cross-sectional approach to establish the associations among negative life events, sleep quality and depression, but it should be noted that a cross-sectional design inhibits causal inference^51^. Longitudinal research is encouraged to provide more compelling evidence for determining the relationships among these factors. Second, the participants were all from Chinese universities, although this has little impact on the research results, it may affect the generalizability of the findings. Diverse community samples should be used in further research to verify the rationality of the results. In addition, studies have demonstrated that the effect of depression can be mitigated by factors such as social support^52^, cognitive hardiness^53^, physical exercise^54^, individual responses^55^ and cultural or socioeconomic factors^56^. It is challenging to comprehensively examine all these factors within a single research project because diverse theoretical constructs and methodologies are used. Therefore, further research endeavors are necessary to explore these variables in detail. Despite these limitations, our study contributes to a deeper understanding of the multifaceted factors that influence mental health among university student populations, offers meaningful insights and underscores the importance of continued research in this critical area.

## Data Availability

The data underlying this article will be shared on reasonable request to the corresponding author.
